# Identification of Critical Regions in Human SAMHD1 Required for Nuclear Localization and Vpx-Mediated Degradation

**DOI:** 10.1371/journal.pone.0066201

**Published:** 2013-07-11

**Authors:** Haoran Guo, Wei Wei, Zhenhong Wei, Xianjun Liu, Sean L. Evans, Weiming Yang, Hong Wang, Ying Guo, Ke Zhao, Jian-Ying Zhou, Xiao-Fang Yu

**Affiliations:** 1 Institute of Virology and AIDS Research, First Hospital of Jilin University, Changchun, Jilin Province, China; 2 Department of Molecular Microbiology and Immunology, Johns Hopkins Bloomberg School of Public Health, Baltimore, Maryland, United States of America; 3 Department of Pathology, Johns Hopkins University School of Medicine, Baltimore, Maryland, United States of America; Shanghai Medical College, Fudan University, China

## Abstract

The sterile alpha motif (SAM) and HD domain-containing protein-1 (SAMHD1) inhibits the infection of resting CD4+ T cells and myeloid cells by human and related simian immunodeficiency viruses (HIV and SIV). Vpx inactivates SAMHD1 by promoting its proteasome-dependent degradation through an interaction with CRL4 (DCAF1) E3 ubiquitin ligase and the C-terminal region of SAMHD1. However, the determinants in SAMHD1 that are required for Vpx-mediated degradation have not been well characterized. SAMHD1 contains a classical nuclear localization signal (NLS), and NLS point mutants are cytoplasmic and resistant to Vpx-mediated degradation. Here, we demonstrate that NLS-mutant SAMHD1 K11A can be rescued by wild-type SAMHD1, restoring its nuclear localization; consequently, SAMHD1 K11A became sensitive to Vpx-mediated degradation in the presence of wild-type SAMHD1. Surprisingly, deletion of N-terminal regions of SAMHD1, including the classical NLS, generated mutant SAMHD1 proteins that were again sensitive to Vpx-mediated degradation. Unlike SAMHD1 K11A, these deletion mutants could be detected in the nucleus. Interestingly, NLS-defective SAMHD1 could still bind to karyopherin-β1 and other nuclear proteins. We also determined that the linker region between the SAM and HD domain and the HD domain itself is important for Vpx-mediated degradation but not Vpx interaction. Thus, SAMHD1 contains an additional nuclear targeting mechanism in addition to the classical NLS. Our data indicate that multiple regions in SAMHD1 are critical for Vpx-mediated nuclear degradation and that association with Vpx is not sufficient for Vpx-mediated degradation of SAMHD1. Since the linker region and HD domain may be involved in SAMHD1 multimerization, our results suggest that SAMHD1 multimerization may be required for Vpx-mediation degradation.

## Introduction

Vpx is a virion-associated viral accessory protein packaged through specific interaction with Gag proteins of HIV-2 and selected SIV lineages [1]–[6]. It is essential for efficient viral replication in macrophages [7]–[10] and dendritic cells [11], [12], promoting the accumulation of viral DNA during reverse transcription [13]–[16]. The Aicardi-Goutières syndrome-related gene product sterile alpha motif (SAM) and HD domain-containing protein-1 (SAMHD1) was recently identified as a potent inhibitor of HIV-1 in myeloid cells and resting CD4+ T cells [17]–[24]. SAMHD1 is a deoxynucleotide triphosphohydrolase and blocks HIV-1 reverse transcription by depleting the intracellular pool of deoxynucleoside triphosphates [18], [21], [25]–[28].

Vpx neutralizes the anti-viral activity of SAMHD1 by promoting its proteasome-dependent degradation. Vpx binds DCAF1 using conserved motifs in helix 1 and helix 3, which in turn recruit other components of the CRL4(DCAF1) E3 ubiquitin ligase [29]–[35] to facilitate SAMHD1 ubiquitination and subsequent degradation [22]–[24], [29], [31]–[34]. Previous researches have indicated that Vpx loads SAMHD1 onto CRL4(DCAF1) E3 ubiquitin ligase, thereby facilitating its subsequent degradation through recognition of C-terminal sequences of SAMHD1 [29], [31], [32], [34]. Consistent with this concept, SAMHD1 mutants with C-terminal truncation are resistant to Vpx-mediated degradation [31], [32], [34]. In addition, the N-terminal region of SAMHD1 contains a classic nuclear localization sequence motif (NLS) which is required for SAMHD1 nuclear targeting and Vpx mediated SAMHD1 degradation [31], [32], [34]. However, the effects of other regions in SAMHD1 on Vpx induced degradation have not been characterized.

In the current study, we observed that deletion of N-terminal regions of SAMHD1 (including NLS) made SAMHD1 mutant proteins again sensitive to Vpx-mediated degradation. Unlike SAMHD1 K11A, these mutants could be detected in the nucleus with nuclear proteins. Thus, SAMHD1 contains an additional nuclear targeting mechanism in addition to the classical NLS. We also identified novel regions in SAMHD1 that are critical for Vpx-mediated degradation but not interaction.

## Materials and Methods

### Plasmid construction

SIVmac239 Vpx-HA in the pCG vector was a gift from Dr. J. Skowronski. pSAMHD1-HA and pSAMHD1-Flag were constructed in our lab as previous described [31]. SAMHD1 muants were constructed from pSAMHD1-HA by PCR based site-directed mutagenesis. To generate an expression vector encoding mCherry-SAMHD1 fusion protein, the SAMHD1-HA fragment was digested with SalI and XbaI and cloned into pmCherry-C1 to generate pmCherry-SAMHD1-HA. pmCherry-SAMHD1K11A-HA was generated by PCR-based site-directed mutagenesis and its sequence confirmed.

### Cell culture and antibodies

HEK293T cells (AIDS Research Reagents Program) were maintained in Dulbecco's modified Eagle's medium (DMEM) with 10% fetal bovine serum and penicillin/streptomycin. All cultured cell lines were maintained at 37°C in a humid atmosphere containing 5% CO2. The following antibodies were used: anti-HA monoclonal antibody (MAb, Covance, MMS-101R), anti-Vprbp (DCAF1, Shanghai Genomics, SG4220-28), anti-FLAG M2 antibody (Sigma, F1804), anti-Myc monoclonal antibody (Covance, MMS-150R), and anti-actin monoclonal antibody (Sigma, A3853).

### Transfection, co-immunoprecipitation, and immunoblotting

DNA transfection was carried out using Lipofectamine 2000 (Invitrogen) according to the manufacturer's instructions. HEK293T cells were harvested at 48 h after transfection, washed twice with cold PBS, and lysed in lysis buffer ( 150 mM Tris, pH 7.5, with 150 mM NaCl, 1% Triton X-100, and complete protease inhibitor cocktail tablets [Roche]) at 4°C for 30 min, then centrifuged at 10,000 g for 30 min. Precleared cell lysates were mixed with anti-HA antibody-conjugated agarose beads (Roche, 190–119) or anti-c-Myc- agaroseaffinity gel (Sigma, A7470), and incubated at 4°C for 3 h or overnight. Samples were then washed eight times with washing buffer (20 mM Tris, pH 7.5 with 100 mM NaCl, 0.1 mM EDTA, and 0.05% Tween 20). The beads were eluted with elution buffer (0.1 M glycine-HCl, pH 2.0). The eluted materials were then analyzed by SDS-PAGE and immunoblotting with the appropriate antibodies as previously described [31].

### Identification of SAMHD1-binding proteins

Expression vectors for SAMHD1-HA and related mutant proteins were transfected into HEK293T cells. SAMHD1-containing complexes were purified from transfected cells by immunoprecipitation using anti-HA affinity matrix (Roche) and analyzed by SDS–PAGE. Protein samples were digested with trypsin. The peptides were separated through a Dionex Ultimate 3000 RELC nano system (Thermo Scientific) with a 75 µm ×15 cm Acclaim PepMap100 separating column (Thermo Scientific) protected by a 2-cm guard column (Thermo Scientific). The mobile phase flow rate was 300 nL/min with 0.1% formic acid in water (A) and 0.1% formic acid 95% acetonitrile (B). The gradient profile was set as follows: 4–35% B for 70 min, 35–95% B for 5 min, and 95% B for 10 min, then equilibration in 4% B for 15 min. MS analysis was performed using an Orbitrap Velos Pro mass spectrometer (Thermo Scientific). The spray voltage was set at 2.2 kV. Orbitrap spectra (AGC 1×106) were collected from 400–1800 m/z at a resolution of 60K, followed by data-dependent HCD MS/MS (at a resolution of 7500, collision energy 45%, activation time 0.1 ms) of the 10 most abundant ions using an isolation width of 2.0 Da. Charge state screening was enabled to reject unassigned and singly charged ions. A dynamic exclusion time of 35 sec was used to discriminate against previously selected ions. Protein identification and label-free quantitation was done using MaxQuant ver. 1.3.0.5, searched against a human protein database ver. 3.87 containing a total of 91,464 entries [36]. The default parameters with a 1% false-discovery rate (FDR) for MaxQuant were used, except for the following: enzyme, trypsin; filter-labeled amino acid, unselected; match between runs, 2 min. Label-free quantification (LFQ) and iBAQ features were selected. The output proteinGroup.txt was used for the quantification.

### Live cell imaging

Plasmid (pEYFP-Nuc [a gift of Dr. T. Inoue], 0.25 µg) and pmCherry-SAMHD1 -HA (2 µg) were transfected into HEK293T cells using PEI Max (Polysciences) according to the manufacturer's protocol. For live cell imaging, HEK293T cells were transfected in 6-well coverslip glass-bottomed cell culture dishes (InVitro Scientific) when the cells were ∼80% confluent, and then visualized after 24 h using a Zeiss LSM510-Meta confocal imaging system equipped with four argon lasers (458-, 477-, 488-, and 514-nm lines), two HeNe lasers (542 and 633 nm), and one diode laser (405 nm). All images were acquired from a 63X objective, and image analysis and manipulation were performed using Zen 2009 software.

## Results

### Identification of novel regions in SAMHD1 that are essential for Vpx-mediated degradation but not for Vpx interaction

**Figure 1:**
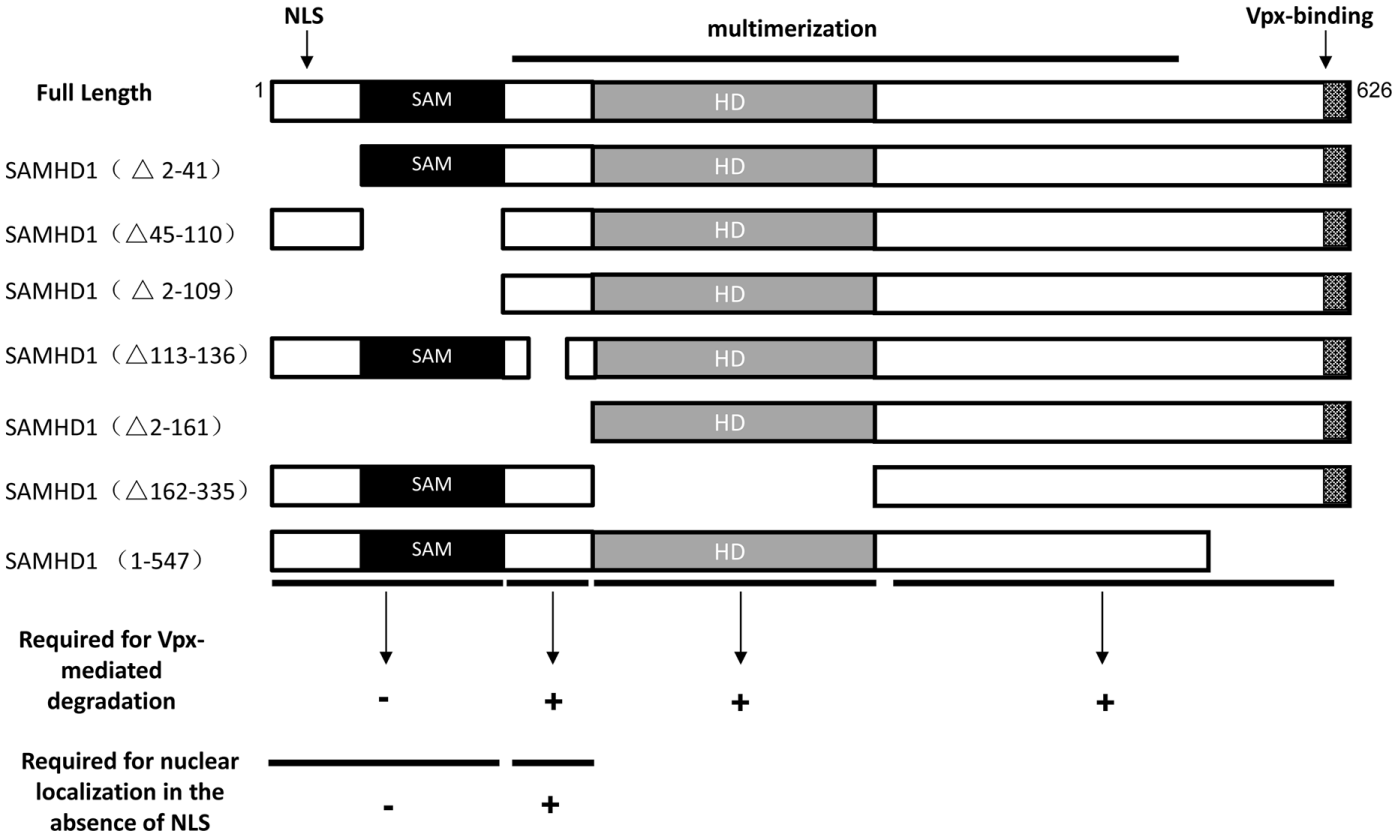
Schematic representation of constructed full-length and truncation variants of SAMHD1. SAMHD1 regions that are dispensable for Vpx-induced degradation are marked (–).

**Figure 2:**
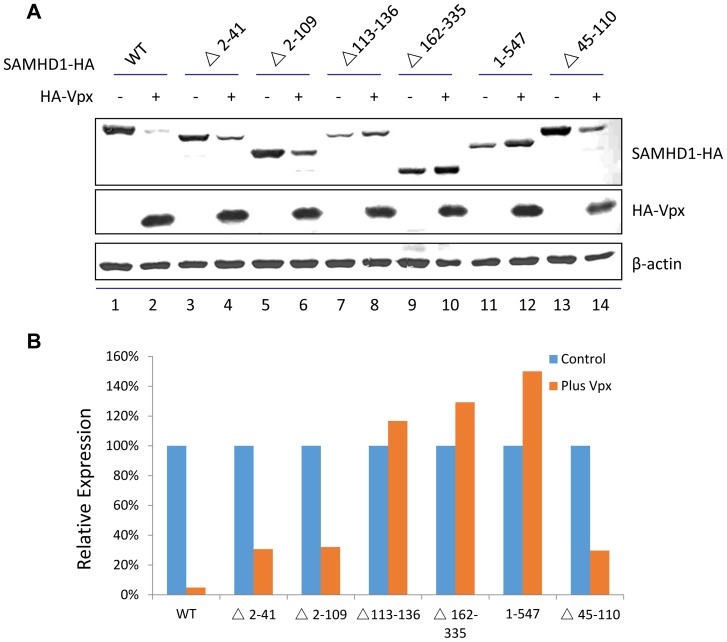
Effects of SAMHD1 region deletion variants on Vpx-induced degradation. (A) Immunoblot of SAMHD1 WT and deletion mutants in the presence and absence of Vpx. HA-tagged SAMHD1 constructs were co-expressed with HA-tagged Vpx or empty vector in 293T cells. Cell extracts were harvested 48 h later and analyzed by SDS-PAGE, followed by immunoblotting to detect SAMHD1-HA and HA-Vpx; β-actin was used as the loading control. (B) The bar graph shows the percentages of relative band intensity for SAMHD1 in the presence of Vpx, vs. in the absence of Vpx.

To further characterize the determinants of SAMHD1 that are involved in Vpx-mediated degradation, we constructed a series of SAMHD1 deletion expression vectors (Fig. 1) covering the N-terminal region (Δ2–41, Δ2–109), SAM domain (Δ45–110), linker region between SAM and the HD domain (Δ113–136), HD domian (Δ162–335), and C-terminal region (1–547). In order to determine whether SAMHD1 variants could be degraded in the presence of Vpx, HA-tagged SAMHD1 mutants were co-expressed with HA-tagged Vpx or empty vector in HEK293T cells. Cell extracts were harvested 48 h after transfection for immunoblot analysis (Fig. 2A), and protein band intensities were quantified using Image J software (Fig. 2B) as previously described [31]. Co-expression of Vpx and SAMHD 1 in transfected HEK293T cells resulted in the depletion of intracellular SAMHD1 (Fig. 2A, lane 2) when compared to the SAMDH1 level in the absence of Vpx (lane 1). However, SAMHD1 mutants containing deletions in the linker region (Δ113–136), HD domain (Δ162–335), or C-terminal region (1–547) were resistant to Vpx-mediated degradation (Fig. 2A, lanes 7–12). In contrast, deletion of the SAM domain (Δ45–110) or other N-terminal regions (Δ2–41 or Δ2–109) resulted in SAMHD1 mutants that were not resistant (Fig. 2A, lanes 3–6,13,14). Surprisingly, the linker domain deletion (Δ113–136) and HD domain deletion (Δ162–335) generated mutant SAMHD1 proteins that were resistant to Vpx-mediated degradation.

**Figure 3:**
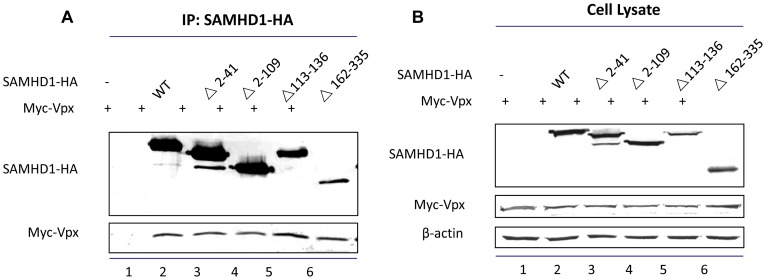
Interaction between SAMHD1 WT/mutant proteins and Vpx. (A) HEK293T cells were co-transfected with a Myc-Vpx expression vector plus control vector (lane 1, VR) or WT SAMHD1-HA (lane 2), or one of the indicated SAMHD1-HA mutants (lanes 3–6). Cell lysates were prepared 48 h after transfection (A) and immunoprecipitated with anti-HA affinity matrix (Roche). The interaction of SIVmac239 Vpx with WT or mutated SAMHD1-HA molecules was detected by immunoblotting with anti-HA antibody to detect Vpx-HFA and anti-Flag antibody to detect SAMHD1-FLAG. (B) Total cell lysates were detected by immunoblotting for indicated proteins.

It is possible that the linker region and HD domain are involved in Vpx binding. To determine whether mutant with deletions within these regions could still interact with SIVmac239 Vpx, we analyzed their interaction with Vpx by co- immunoprecipitation experiments. myc-tagged Vpx and HA-tagged SAMHD1 wild-type or mutants were co-expressed in HEK293T cells, and cell lysates were subjected to co-immunoprecipitation using an anti-HA antibody conjugated to agarose beads at 48 h after transfection, as previously described [37]. The anti-HA affinity matrix (Roche) immunoprecipitated wild-type HA-tagged SAMHD1 as well as the deletion mutants from cell lysates of transfected HEK293T cells (Fig. 3A). As expected, although myc-Vpx was expressed efficiently in HEK293T cells (Fig. 3B), it was not immunoprecipitated by the anti-HA affinity matrix in the absence of SAMHD1 (Fig. 3A, lane 1), confirming the specificity of the assay system. In repeated experiments, we saw co-precipitation of myc-Vpx with SAMHD1 N-terminal region deletions (Δ2–41 or Δ2–109), a linker domain deletion (Δ113–136), and an HD domain deletion (Δ162–335) in addition to wild-type SAMHD1 (Fig. 3A). SAMHD1(Δ162–335) may even have a slight increased ability to interact with Vpx. Thus, the linker region and HD domain deletions compromised Vpx-mediated SAMHD1 degradation but not the interaction between Vpx and SAMHD1.

### Deletion of a well-characterized nuclear targeting signal resulted in SAMHD1 mutant proteins in the nucleus that were sensitive to Vpx-mediated degradation

**Figure 4:**
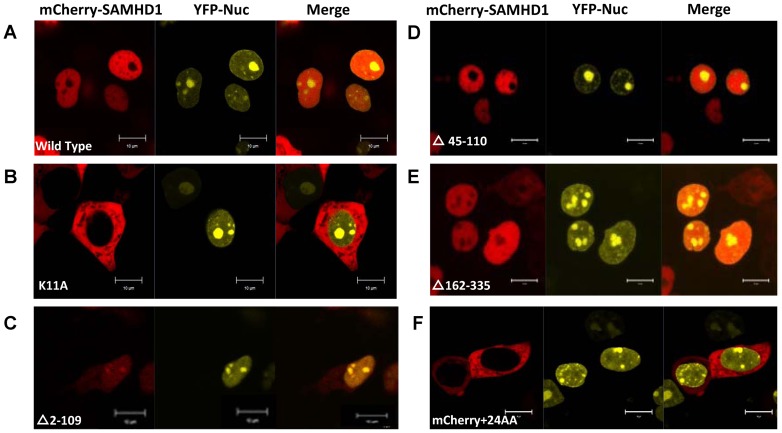
Cellular localization of mCherry-tagged SAMHD1 and SAMHD1 mutant proteins using live cell imaging. (A)–(F) Plasmid pEYFP-Nuc (Clontech) and pmCherry-SAMHD1 WT or indicated variants were co-transfected into HEK293T cells using PEI Max (Polysciences). For live cell imaging, HEK293T cells were transfected in 6-well coverslip glass-bottomed cell culture dishes (InVitro Scientific) when the cells were ∼80% confluent, and then visualized after 24 h using a Zeiss LSM510-Meta confocal imaging system equipped with four argon lasers (458-, 477-, 488-, and 514-nm lines), two HeNe lasers (542 and 633 nm), and one diode laser (405 nm). All images were acquired from a 100X objective, and image analysis and manipulation were performed using Zen 2009 software.

**Figure 5:**
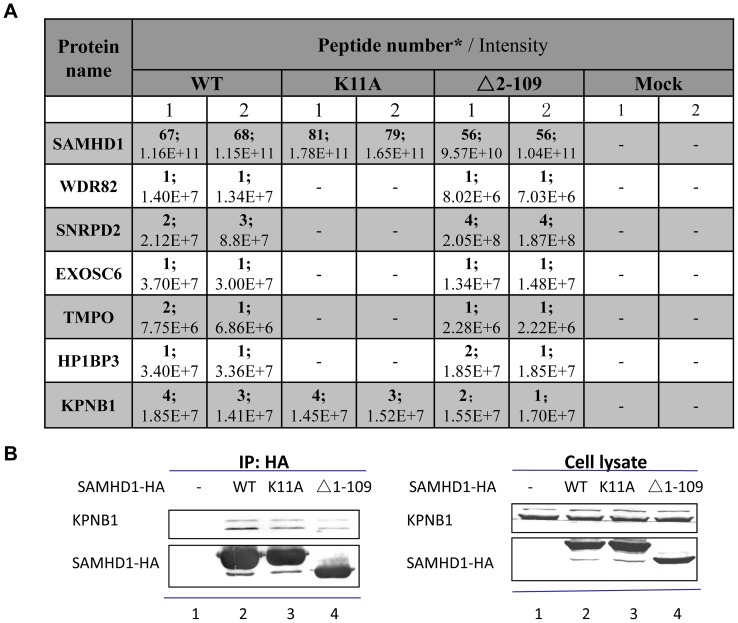
Loss of binding of cytoplasmic SAMHD1 K11A to SAMHD1-associated nuclear proteins. (A) HEK293T cells were transfected with SAMHD1 WT, K11A, Δ2–109, or empty vector control, respectively. Co- immunoprecipitation was carried out 48 h later by using anti-HA affinity matrix (Roche). Protein samples were analyzed by mass-spec as described in the Methods. (B) Interaction between SAMHD1 WT/mutant proteins and KPNB1. HEK293T cells were transfected with a control vector (lane 1, VR) or WT SAMHD1-HA (lane 2), or one of the indicated SAMHD1-HA mutants (lanes 3–4). Cell lysates were prepared 48 h after transfection and immunoprecipitated with anti-HA affinity matrix (Roche). The interaction of KPNB1 with WT or mutated SAMHD1-HA molecules was detected by immunoblotting with anti-HA antibody to detect SAMHD1-HA. A specific anti-KPNB1 was used to detect endogenous KPNB1.

Recently, we and others identified a classical nuclear localization signal (NLS) ^11^KRPR^14^ in human SAMHD1 that is required for nuclear localization and Vpx-induced degradation of SAMHD1 [31], [32], [34]. Surprisingly, deletion of this NLS sequence plus surrounding amino acids (2–41 and 2–109) resulted in mutant SAMHD1 proteins that became sensitive to Vpx-mediated degradation (Fig. 2). If nuclear localization of SAMHD1 is important for Vpx-mediated degradation, SAMHD1 mutants (Δ2–41 or Δ2–109) may have recovered some nuclear targeting ability. To examine the cellular localization of SAMHD1 proteins and their relationship to Vpx-mediated degradation, we cloned the wild-type or mutant SAMHD1 coding region into pmCherry-C1 vectors (Clontech Laboratories) to express fusion proteins for live cell imaging. HEK293T cells were transfected with pEYFP-Nuc for nuclear visualization (Clontech Laboratories) and pmCherry-SAMHD1-HA in 6-well coverslip dishes using PEI Max (Polysciences). Cells were visualized 24 h after transfection using a Zeiss LSM510-Meta confocal imaging system as previously described [38]. All images were acquired from a 63X objective, and image analysis and manipulation were performed using Zen 2009 software. As expected, wild-type SAMHD1 was detected mainly in the nucleus (Fig. 4A). Interestingly, we found that even without the NLS, SAMHD1 mutant (Δ2–109) could be observed in the nucleus of the transfected cells (Fig. 4C). In contrast, SAMHD1K11A was totally localized to the cytoplasm (Fig. 4B). Deletion of the HD (Δ162–335) and SAM (Δ45–110) domains did not affect nuclear accumulation of these mutant SAMHD1 proteins (Fig. 4D and E). Consistent with the idea that SAMHDΔ2–109 is in the nucleus, we have observed (Fig. 5A) an interaction of wild-type SAMHD1 and SAMHDΔ2–109, but not SAMHD1K11A, with known nuclear-resident proteins (http://www.uniprot.org/) such as WDR82 [39], TMPO [40], and SNRPD2 [41]. Interestingly, our mass-spec results (Fig. 5A) also showed that wild-type SAMHD1, SAMHDK11A and SAMHDΔ2–109 could bind to karyopherin-β1 (KPNB1). Co-IP and immunoblot analysis confirmed this interaction (Fig. 5B).

**Figure 6:**
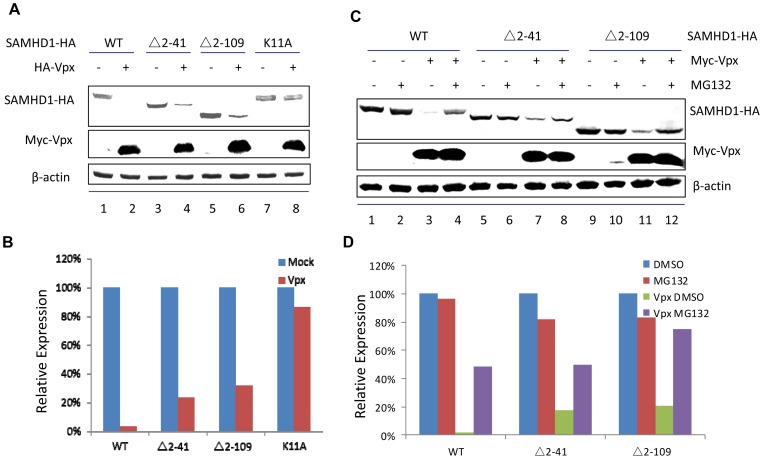
Partial nuclear accumulation of SAMHD1 N-terminal deletion mutants, which specially bind to nuclear proteins and increase the sensitivy to Vpx induced degradation. (A) HA-tagged SAMHD1 WT or mutants were co-expressed with HA-tagged Vpx or empty vector in HEK293T. Cell extracts were harvest 48 h later and analyzed by SDS-PAGE, followed by immunoblotting to detect SAMHD1-HA and HA-Vpx, with β-actin as loading control. (B) The bar graph shows the percentages of the relative band intensities for SAMHD1 in the presence of Vpx vs. in the absence of Vpx. (C) HA-tagged SAMHD1 WT or mutants were co-expressed with HA-tagged Vpx or empty vector in HEK293T in the absence or presence of MG132. Cell extracts were harvest 48 h later and analyzed by SDS-PAGE, followed by immunoblotting to detect SAMHD1-HA and HA-Vpx, with β-actin as loading control. (D) The bar graph shows the percentages of the relative band intensities for SAMHD1 in the presence of Vpx vs. in the absence of Vpx plus or minus MG132.

Further comparison revealed that SAMHD1 mutants (Δ2–41 and Δ2–109) with partially recovered nuclear targeting were indeed more sensitive to Vpx-induced degradation than was the SAMHD1K11A mutant (Fig. 6A). Under conditions in which >60% of the SAMHD1 mutants (Δ2–41 and Δ2–109) could be degraded in the presence of Vpx, >90% of SAMHD1K11A remained undegraded (Fig. 6B). The Vpx-mediated degradation of SAMHD1 mutants (Δ2–41 and Δ2–109) could be inhibited by proteasome inhibitor MG132 (Fig. 6C, 6D). These results further demonstrated the important role of SAMHD1 nuclear localization in Vpx-mediated degradation.

### Cytoplasmic mutant SAMHD1 K11A could be translocated into the nucleus in the presence of wild-type SAMHD1 and became sensitive to Vpx-mediated degradation

**Figure 7:**
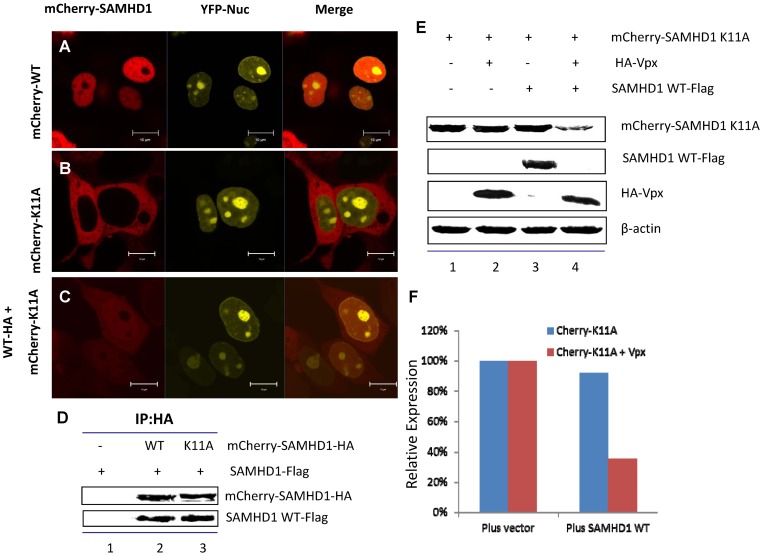
WT SAMHD1 relocates cytoplasm-localized mutants of SAMHD1K11A to the nucleus and facilitates their Vpx-induced degradation. (A)–(C) Wild-type SAMHD1-HA partially translocated pmCherry-SAMHD1 K11A to the nucleus. pmCherry-SAMHD1-HA WT and/or K11A with pEYFP-Nuc were co-transfected into HEK293T cells using PEI Max. Live cells were imaged at 24 h post-transfection. (D) Interaction between wild-type SAMHD1 and K11A. pSAMHD1 WT-Flag was co-transfected with pmCherry-SAMHD1-HA WT or K11A. Transfected cells were harvested after 48 h and incubated in lysis buffer for 30 min. Cell lysates were added to an anti-HA affinity matrix (Roche). Eluted protein samples were detected by immunoblotting with anti-HA and anti-Flag antibody to detect mCherry-SAMHD1 and SAMHD1-Flag, respectively. (E) Immunoblotting demonstrates that pmCherry-SAMHD1 K11A became sensitive to Vpx-mediated degradation when co-expressed with SAMHD1 WT. HEK293T cells were co-transfected with HA-Vpx and pmCherrySAMHD1 K11A and/or pSAMHD1-Flag wild-type. Cell extracts were harvested 48 h later and analyzed by SDS-PAGE, followed by immunoblotting to detect SAMHD1-HA and HA-Vpx. β-actin was used as the loading control.

If nuclear targeting of SAMHD1 is critical for Vpx-mediated degradation, targeting SAMHD1K11A to the nucleus should restore its sensitivity to Vpx-mediated degradation. The crystal structure and biochemical analysis of SAMHD1 revealed that SAMHD1 has the potential to form oligomers [19], [27], [28], [42]. However, whether the cytoplasmic SAMHD1K11A variant can interact with wild-type SAMHD1 and be translocated to the nucleus has not been tested. To examine this issue, we co-expressed a pmCherry-SAMHD1K11A mutant and wild-type SAMHD1-HA in HEK293T cells for live cell imaging. As expected, pmCherry-SAMHD1 (wild-type) was detected in the nucleus (Fig. 7A) and pmCherry-SAMHD1K11A in the cytoplasm (Fig. 7B). However, in HEK293Tcells co-expressing pmCherry-SAMHD1K11A and wild-type SAMHD1-HA, red fluorescence (pmCherry-SAMHD1K11A) could be detected in the nucleus (Fig. 7C). Since wild-type SAMHD1-HA proteins do not generate fluorescence, these results indicate that pmCherry-SAMHD1K11A proteins were translocated into nucleus in the presence of non-fluorescent wild-type SAMHD1-HA. Interaction of SAMHD1K11A mutants with wild-type SAMHD1 was detected by co-immunoprecipitation experiments (Fig. 7D). We have also observed that mCherry-tagged SAMHD1K11A was sensitive to Vpx-mediated degradation in the presence of wild-type SAMHD1 (Fig. 7E).

## Discussion

The C-terminal region of human SAMHD1 has been identified as a Vpx-binding domain and therefore as important for Vpx-mediated degradation [20], [29], [31], [34], [43]. In the current study, we have identified additional important regions in SAMHD1 that are required for Vpx-mediated degradation. Our results with N-terminal deletions of different lengths suggested that the N-terminal sequences, including the SAM domain, are not essential for Vpx recognition or Vpx-mediated degradation of SAMHD1. However, the SAMHD1 linker region and HD domain deletion mutants were resistant to Vpx-triggered degradation. Interestingly, the SAMHD1 linker region and HD domain deletion mutant proteins could still interact with Vpx. Thus, binding to Vpx is necessary but not sufficient for Vpx-mediated degradation by SAMHD1.

The phenomenon we have observed – that substrate receptor-CRL binding is not sufficient for target protein degradation – is not without precedent. HIV-1 Vif recruits Cul5-based CRL and targets APOBEC3G for polyubiquitination and subsequent degradation. However, APOBEC3G mutants that are Vif-binding-competent but resistant to Vif-mediated degradation have been described [44], [45]. It is possible that the linker region and HD domain of SAMHD1 are required for proper folding and ubiquitination. The linker region and HD domain may be involved in SAMHD1 multimerization. Thus, our study raised the question of whether SAMHD1 multimerization is required for Vpx-mediation degradation.

The data presented here, and those published recently by others, indicate that SAMHD1 nuclear accumulation is a dominant factor for Vpx-induced degradation. The classical NLS(KRPR) is critical for nuclear localization of full-length SAMHD1. Surprisingly, SAMHD1 N-terminal deletion variants (Δ2–41, Δ2–109) showed increased nuclear location when compared to the SAMHD1 K11A mutant and became more sensitive to Vpx-mediated degradation. Previous studies have clearly shown that passive diffusion of proteins into the nucleus is reasonably effective only for proteins of <40 kDa [46]–[49]. The estimated ∼80-kDa size of mCherry-SAMHD1Δ2–41 (843aa) and mCherry-SAMHD1Δ2–109 (775aa) are well above upper limit of the cut-off imposed by the diameter of the nuclear pores for passive nuclear diffusion. Furthermore, mCherry itself with an additional 24aa became mostly cytoplasmic (Fig. 4F). These data suggest that SAMHD1 has an additional nuclear targeting mechanism in addition to the classical NLS. Interestingly, NLS minus SAMHD1 could still bind to karyopherin-β1. Future study will be required to determine whether karyopherin-β1 is involved in SAMHD1 nuclear targeting.

If SAMHD1 has an additional nuclear targeting mechanism, why is full-length SAMHD1K11A cytoplasmic but SAMHD1 (Δ2–41 and Δ2–109) mutants targeted to the nucleus? At least three models could be proposed to explain these observations: (1) SAMHD1 has another nuclear targeting mechanism in addition to NLS (KRPR). The additional nuclear targeting mechanism is only revealed when the N-terminal region is deleted. (2) The N-terminal region of SAMHD1 contains a dominant cytoplasmic retention signal that can only be suppressed by the NLS (KRPR). N-terminal deletions (Δ2–41 and Δ2–109) destroyed the cytoplasmic retention signal. Thus, SAMHD1K11A is cytoplasmic, yet SAMHD1 (Δ2–41 and Δ2–109) mutants could still be detected in the nucleus. (3) The N-terminal region of SAMHD1 contains a nuclear export signal that is normally suppressed by the NLS (KRPR). N-terminal deletions (Δ2–41 and Δ2–109) destroyed the nuclear export signal to allow the nuclear localization of SAMHD1(Δ2–41 and Δ2–109) mutants. Thus, SAMHD1 may contain two nuclear localization signals and one cytoplasmic retention or nuclear export signal. These various nuclear targeting and nuclear export/cytoplasmic retention signals may play important regulatory roles in the cellular function of SAMHD1.
